# Uric acid variability at midlife as an independent predictor of coronary heart disease and all-cause mortality

**DOI:** 10.1371/journal.pone.0220532

**Published:** 2019-08-05

**Authors:** Chagai Grossman, Ehud Grossman, Uri Goldbourt

**Affiliations:** 1 Department of Internal Medicine F and the Rheumatology Unit, The Chaim Sheba Medical Center, Tel-Hashomer, affiliated to Sackler Faculty of Medicine, Tel-Aviv University, Tel-Aviv, Israel; 2 Department of Internal Medicine D and the Hypertension Unit, The Chaim Sheba Medical Center, Tel-Hashomer, affiliated to Sackler Faculty of Medicine, Tel-Aviv University, Tel-Aviv, Israel; 3 Department of Epidemiology and Preventive Medicine, School of Public Health, Sackler Faculty of Medicine, Tel-Aviv University, Tel-Aviv, Israel; Shanghai Institute of Hypertension, CHINA

## Abstract

**Background:**

Serum uric acid (SUA) has long been associated with cardiovascular disease. Variability of serum uric acid (SUA) has seldom been examined in association with long-term morbidity and mortality. Therefore, we aimed to investigate the association between SUA variability and long-term all-cause and specific-cause mortality.

**Methods:**

Among 10,059 men, aged 40–65, tenured civil servants and municipal employees in Israel, 8822 participants who were examined in 1963, 1965 and 1968 had assessment of diabetic and coronary morbidity status and SUA levels. We conducted analysis examining whether the standard deviations (SD) of Z-scores of SUA across study visits predicted coronary heart disease (CHD) and mortality. Hazard ratios (HR) associated with the SD of SUA-Z were calculated for stroke, CHD mortality and all-cause mortality associated with quartiles of the above variability.

**Results:**

Multivariate analysis of 18-year CHD mortality yielded a significant association with the 1963–1968 SD of SUA-Z with age adjusted HR of CHD mortality of 0.97 (95% CI, 0.8–1.19), 1.05 (95% CI, 0.87–1.28) and 1.37 (95% CI, 1.15–1.65) for quartiles 2 to 4 respectively). The results of all-cause mortality similarly and strongly indicated increasing age-adjusted mortality risk with increasing SD of SUA-Z: HR = 1.08 (95% CI, 0.97–1.21), 1.15 (1.03–1.28) and 1.37 (1.23–1.51). No association was observed between the SD of SUA-Z and stroke mortality.

**Conclusion:**

In this cohort of tenured male workers, with diverse occupations, higher variability of SUA measurement taken over 5 years was clearly predictive of 18-year CHD and all-cause mortality, above and beyond the SUA levels proper.

## Background

Serum uric acid (SUA) has long been associated with dyslipidemia, diabetes, hypertension, coronary calcifications and renal failure [1–3]. It has been suggested that elevated SUA may be associated with mortality in high risk patients with asymptomatic carotid atherosclerotic disease, acute myocardial infarction, heart failure, diabetes mellitus and hypertension [4–7]. The role of SUA as an independent risk factor for CV mortality is questionable. It is not clear whether SUA has a causal role in the development of CV disease and death or whether the association is circumstantial. It is possible that SUA correlates with CV risk factors and may reflect an underlying CV disease [8–14]. Data regarding the association between SUA and long-term mortality are mainly based on a single baseline SUA measurement. Recently, we have shown an association between baseline SUA and long-term mortality from stroke, coronary heart disease (CHD) and all causes mortality [15]. These associations were based on a single baseline SUA measurement. It has been suggested that elevated SUA levels may blunt renal auto-regulation of blood pressure and cause endothelial dysfunction [16, 17]. If SUA is related to blood pressure levels, then SUA levels may fluctuate in parallel to blood pressure fluctuations. Several studies have shown an association between blood pressure variability and CV morbidity and mortality [18, 19]. We therefore designed this study to further evaluate the association between the visit-by-visit variability of SUA levels and CV and all-cause mortality.

## Methods

The current study is based on the IIHD study, which was conducted as a collaborative project of the National Heart and Lung Institute, NIH, USA, the Israel Civil Service Commission and the Hadassah Medical Organization, at the beginning of the 1960 sec, a time when Ethical Review Boards did not yet exist in Israel. However, all participants had given their oral consent to take part in the study upon their recruitment in 1963 following explanations regarding the study objectives and the long-term follow up. In addition, the Tel Aviv University Ethical Review Board approved of the linkage of the IIHD database with the Israel Population Registry. Furthermore, Prof. Goldbourt, an author in this publication, is legally responsible for the IIHD study database and has approved its use for the purpose of this study.

### Study participants

The original cohort included 10,059 individuals, who were recruited by stratified sampling of civil servants and municipal employees in 1963, based on the following inclusion criteria: 1. Men aged 40 years or older. 2. Work place limited to the three largest urban areas in Israel. The sample size was aimed at obtaining sufficient number of participants from six areas of birth which were proportional to the Israeli male population of this age. Participants underwent clinical, dietary, psychological and blood biochemical evaluations in 1963, 1965 and 1968. Further details of the study have previously been described [20, 21]. Patients with missing measurement of SUA in one of the evaluations (n = 1237) were not included in the study. Thus, the present analysis includes 8822 participants who had 3 measurements of SUA and baseline assessment of diabetic and coronary morbidity status.

### Mortality data

Follow-up since the last evaluation on 1968 lasted 18 years. Data regarding death was derived from the Israeli Mortality Registry. The underlying cause of death was documented on the basis of case-by-case determinations by a review panel until 1970 and by the use of International Classification of Diseases (ICD) codes thereafter.

### Uric acid measurements

Blood for measurement of SUA levels was drawn on each evaluation. SUA was measured by Fister's adaptation of the colorimetric method using phosphotungstic acid in the presence of cyanide and urea. All determinations were performed in duplicate and the mean result of the two tests was used for analysis. SUA variability was defined as the standard deviations (SD) of Z-scores of SUA across study visits.

### Risk factor assessment

Blood pressure (BP) was measured with a standard mercury sphygmomanometer, taken twice in the supine position, with a time interval of 15–30 between both measurements. The second measurement was used for the analysis. Non-fasting cholesterol was measured using the Anderson and Keys modification of the Abel method [22]. Cigarette smoking was self-reported, and was classified as ever-smoked or not. Additional variables included diabetes mellitus (DM), body mass index (BMI) and history of CHD, defined as confirmed angina pectoris, documented hospitalization for myocardial infarction, or electrocardiographic pattern of an old infarction.

### Statistical analysis

We conducted analysis examining whether the standard deviations (SD) of Z-scores of SUA across study visits predicted CHD- and stroke- as well as all-cause mortality. SUA-Z was defined as the difference between the individual SUA and the mean of SUA, divided by the standard deviation (SD) for the pertinent examination, namely separately for the 1963, 1965 and 1968 means and SD. Hazard ratios (HR) were calculated applying Cox proportional hazard models. The proportional hazard assumption was assessed applying Schoenfeld residuals. HR associated with the SD of SUA-Z were calculated for 18-year stroke-, CHD- and all-cause mortality associated with quartiles of the above variability. The lower quartile served as the referent, adjusting for age. A subsequent model adjusted additionally for the baseline value of SUA as well as for baseline frequency of diabetes mellitus and CHD. Stratified rate ratios were calculated using a Mantel-Haenszel-type method. This was used to carry out trend tests for an increment of one quartile of SD of SUA-Z. The extent by which including the visit-by-visit SUA variability as a predicting factor of mortality adds to the prediction of all-cause and CHD mortality was estimated by Harrell's C concordance index and by Somers' D index. Statistical analysis was carried out using the STATA statis-tical package, version 15 (STATA, College Station, TX).

Significance was considered when the p value was < 0.05.

## Results

### Subject characteristics

A total of 8822 men were included in the analysis. Participants were divided according to quartiles of SUA variability during the years 1963–1968. Baseline characteristics are presented in Table 1. About two thirds of the participants had smoked at any time. Participants had a mean BMI of 25.7 ± 3.3 Kg/m^2^, their baseline SUA was relatively low and the baseline rates of diabetes mellitus and CHD were low. Participants in the 4^th^ quartile had higher body mass index and initial SUA levels.

**Table 1:** Baseline clinical characteristics according to quartiles SD of serum uric acid variability.

	Total study population	Quartile 1 (n = 2203)	Quartile 2 (n = 2207)	Quartile 3 (n = 2207)	Quartile 4 (n = 2205)	P value
SUA 1963 (mg/dL)	4. 75 ± 0.96	4.54 ± 0.81	4.67 ± 0.82	4.73 ± 0.88	5.02 ± 1.18	0.0005>
SUA 1965 (mg/dL)	5.17 ± 1.05	4.96 ± 0.89	5.08 ± 0.91	5.14 ± 0.86	5.50 ± 1.27	
SUA 1968 (mg/dL)	5.25 ± 1.08	5.03 ± 0.93	5.15 ± 0.91	5.23 ± 1.01	5.61 ± 1.34	
SUA variability range	0.050–3.148	0.050–0.191	0.191–0.310	0.310–0.464	0.464–3.148	
Age (years)	49.3 ± 6.9	48.7± 6.6	48.9±6.6	49±6.6	49.7±7	0.0005>
BMI (kg/m^2^)	25.7 ± 3.3	25.4 +-3.1	25.7+-3.3	25.7+-3.3	26.1+-3.3	0.0005>
SBP (mmHg)	135± 21	133 ± 19	134±20	134±20	138±22	0.0005>
DBP (mmHg)	84±11	83±10	84±11	83±11	85±12	0.0005>
Cholesterol (mg/dL)	209±40	208 ±39	209±40	209±40	210±41	0.69
Ever smoked (%)	68.6	67.3	69.7	67.0	68.0	0.17
Diabetes (%)	4.8	3.9	3.9	4.9	5	0.16
CHD (%)	4.4	3	3.8	3.9	5.3	0.07

SD, standard deviation; BMI, body mass index; SBP, systolic blood pressure, DBP, diastolic blood pressure; CHD, coronary heart disease, SUA, serum uric acid.

### Association between serum uric acid variability and long-term mortality

**Fig 1:**
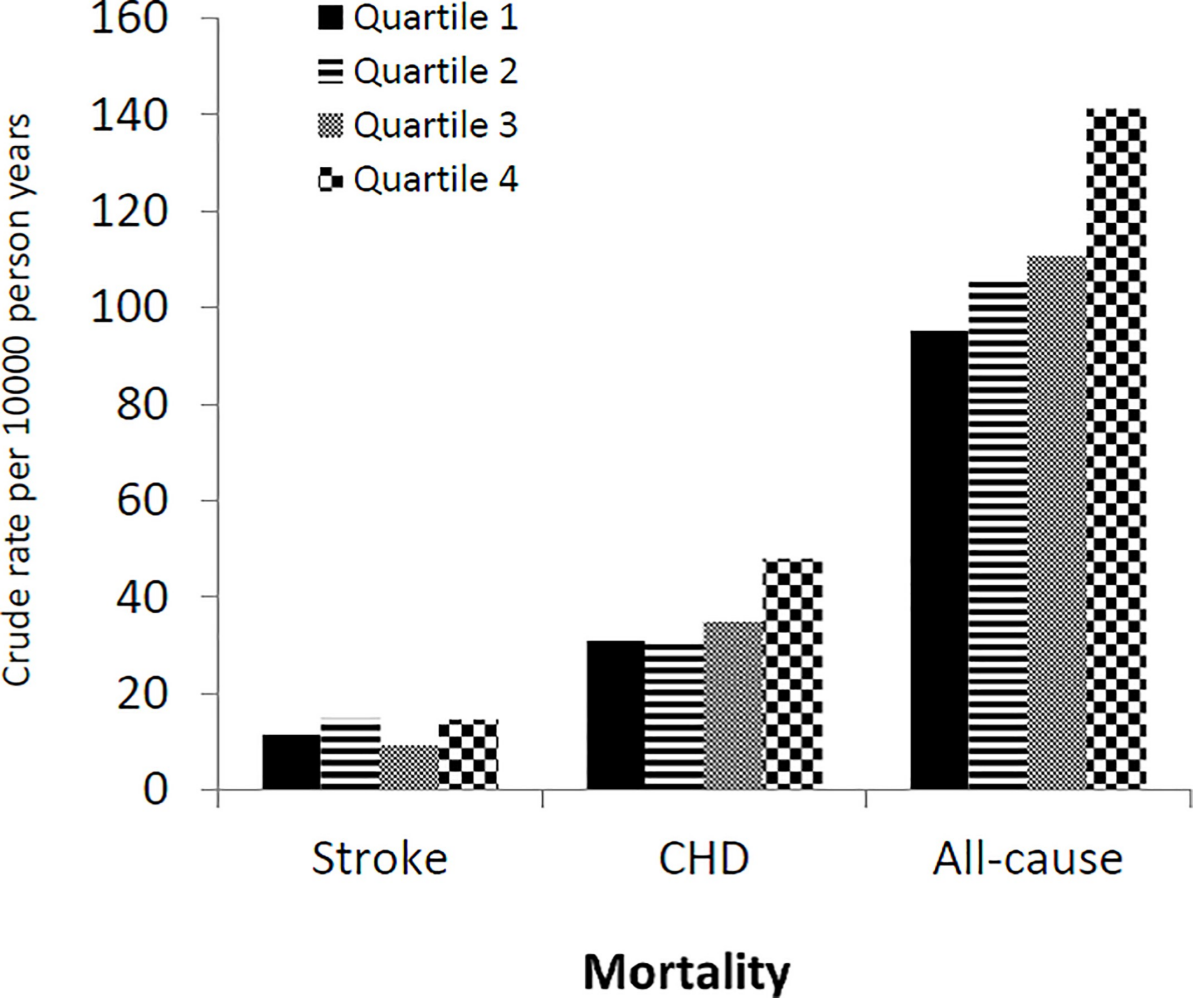
Crude mortality from stroke, coronary heart disease (CHD) and all-cause according to quartile of uric acid variability.

During the follow up 2893 subjects died, 830 of them died from CHD and 292 died from stroke. Rates of all-cause mortality and mortality from stroke and CHD per 10000 person years are given in Fig 1. Stroke mortality was not associated with SUA variability (Table 2). However, the rate of CHD mortality and all-cause mortality was higher in the 4^th^ quartile of the SUA variability (Fig 1).

**Table 2:** Adjusted Hazard ratio for stroke mortality by quartiles of SD of serum uric acid variability.

	HR	95% CI	p-value
**Mode 1**			
Quartile 2	1.28	0.94–1.73	0.12
Quartile 3	0.77	0.54–1.08	0.13
Quartile 4	1.15	0.84–1.57	0.38
**Model 2**			
Quartile 2	1.28	0.94–1.73	0.12
Quartile 3	0.76	0.54–1.08	0.13
Quartile 4	1.14	0.83–1.56	0.42
**Model 3**			
Quartile 2	1.24	0.91–1.68	0.17
Quartile 3	0.72	0.51–1.02	0.06
Quartile 4	0.97	0.71–1.34	0.86

HR = hazard ratio, CI = confidence interval, SD = standard deviation, CHD =

coronary heart disease

Model 1: Age adjusted. Model 2: Adjusted for age and baseline uric acid

Model 3: Adjusted for age, systolic blood pressure, baseline uric acid, diabetes mellitus and ischemic heart disease.

**Fig 2:**
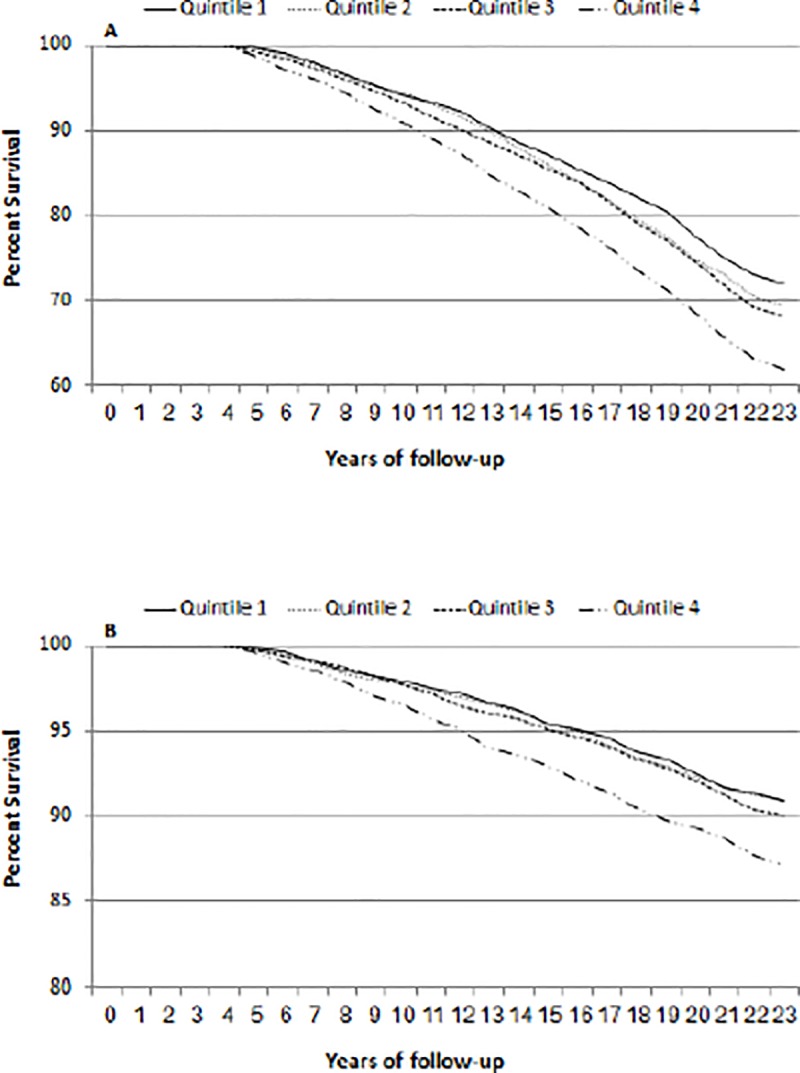
Kaplan-Meier survival curve for all-cause mortality (A) and CHD mortality (B) as a function of quartile of uric acid variability. (P < 0.01).

Multivariate analysis of 18-year CHD mortality yielded a significant association between SUA variability during the years 1963–1968 (Table 3). Similarly, the results for all-cause mortality showed increasing age-adjusted mortality with increasing SUA variability (Table 4). Kaplan-Meier survival curves for all-cause mortality and CHD mortality as a function of SUA vaiability are presented in Fig 2. In tests for trend, the average risk ratio associated with a rise in one quartile was 1.14 (95%CI; 1.06–1.23) for CHD mortality and 1.10 (95%CI; 1.06–1.23). Sensitivity analysis, incorporating the last (1968) SUA levels assessed, rather than the 1963 ones, yielded virtually identical HR. Estimating the differentiation advantage associated with inclusion of the visit-by-visit SUA variability yielded the following results: Harrell's C concordance index rose from 0.703 for all-cause mortality and 0.718 for CHD mortality, excluding the above variability, to 0.720 and 0.743 for all-cause and CHD mortality, respectively. Corresponding results for the Somers' D index yielded estimated rises from 0.406 and 0.436 to 0.440 and 0.486, respectively.

**Table 3:** Adjusted Hazard ratio for CHD mortality by quartiles of SD of serum uric acid variability.

	HR	95% CI	p-value
**Model 1**			
Quartile 2	0.99	0.82–1.21	0.95
Quartile 3	1.12	0.92–1.35	0.26
Quartile 4	1.43	1.20–1.72	<0.0005
**Model 2**			
Quartile 2	0.98	0.81–1.20	0.87
Quartile 3	1.09	0.90–1.33	0.36
Quartile 4	1.35	1.12–1.62	0.002
**Model 3**			
Quartile 2	0.98	0.81–1.19	0.80
Quartile 3	1.03	0.85–1.25	0.71
Quartile 4	1.27	1.06–1.52	0.007

HR = hazard ratio, CI = confidence interval, SD = standard deviation, CHD = coronary heart disease. Model 1: Age adjusted. Model 2: Adjusted for age and baseline uric acid. Model 3: Adjusted for age, Systolic blood pressure, baseline uric acid, diabetes mellitus and ischemic heart disease.

**Table 4:** Adjusted Hazard ratio for all-cause mortality by quartiles of SD of serum uric acid variability.

	HR	95% CI	p-value
**Mode 1**			
Quartile 2	1.09	0.98–1.21	0.13
Quartile 3	1.15	1.03–1.28	0.013
Quartile 4	1.37	1.23–1.52	<0.0005
**Model 2**			
Quartile 2	1.08	0.97–1.21	0.14
Quartile 3	1.14	1.02–1.27	0.02
Quartile 4	1.33	1.20–1.48	<0.0005
**Model 3**			
Quartile 2	1.07	0.96–1.20	0.16
Quartile 3	1.10	0.99–1.23	0.07
Quartile 4	1.28	1.15–1.42	<0.0005

HR = hazard ratio, CI = confidence interval, SD = standard deviation, CHD = coronary heart disease. Model 1: Age adjusted. Model 2: Adjusted for age and baseline uric acid. Model 3: Adjusted for age, systolic blood pressure, baseline uric acid, diabetes mellitus and ischemic heart disease.

## Discussion

In the present study, we showed for the first time that variability in SUA levels is associated with long term CHD and all-cause mortality. It is well established that SUA is associated with cardiovascular risk factors, including hypertension, hyperlipidemia, obesity and renal failure [1, 2]. It has also been shown that SUA is associated with CHD [13, 23, 24]. The role of SUA as an independent risk factor for CVD and death is controversial. Several studies have investigated this issue, revealing conflicting results. Culleton et al. found that among women, SUA was predictive of CHD, death from CV disease and death from all causes. However, after adjustment for CV risk factors, SUA was no longer associated with CHD, death from CV disease, or death from all causes. These findings indicate that SUA does not have a causal role in the development of CHD or CV mortality [9]. Other studies have demonstrated that SUA levels are an independent predictor of CV mortality [10–13].

In the present study, we aimed to investigate the association of SUA variability with long-term all-cause and specific-cause mortality. The meaning of risk factors variability has been previously investigated with regard to several parameters. Blood pressure variability was associated with CV morbidity and mortality [18, 19, 25]. Further studies have demonstrated that glycemic and blood pressure variability may be independent risk factors for the development of albuminuria and for decreased glomerular filtration rate (GFR) in patients with type 2 DM [26, 27]. Fluctuations in body weight are associated with coronary death [28]. A recent study showed an association between body weight variability and CV events in patients with coronary events [29]. Apparently it seems that fluctuations in hemodynamic and metabolic parameters are associated with poor outcome. A possible explanation for the association of variation in blood pressure and body weight with increased CV risk is that the need to accommodate to blood pressure or body weight fluctuations requires energy recruitment through activation of the sympathetic nervous system and the renin angiotensin system.

In the present study we have shown that SUA variability between the years 1963–1968 was associated with CHD mortality and all-cause mortality beyond and above SUA levels properly.

Data regarding the significance of SUA variability are scarce. Ceriello et al. investigated the impact of variability of HbA1c, systolic and diastolic blood pressure, cholesterol, triglycerides and SUA levels on developing chronic kidney disease among diabetic patients. They found that high variability in all the aforementioned parameters predicted the decline in GFR. High variability in SUA levels conferred the highest risk of decline in GFR [30]. Microalbuminuria and reduced kidney function serve as surrogate markers for CV disease in diabetes, as both are independent risk factors for CV events among patients with type 2 DM [31]. These findings are consistent with our findings that SUA variability is an independent risk factor for CV events. It is unclear whether SUA is an independent CV risk factor or reflects an underlying CV disease [9–14]. If SUA was an independent risk factor for CV events, then thiazide diuretics, which increase SUA, should increase, and drugs, which lower SUA, should decrease CV morbidity and mortality. Unfortunately, there is no evidence for the detrimental effects of diuretic and beneficial effects of SUA lowering agents on CV outcomes. Thus, it is more likely that SUA reflects an underlying CV disease. If we assume that SUA is a marker of metabolic and hemodynamic changes, then we can understand why SUA variability is associated with CHD and all-cause mortality. Several additional explanations for the effect of SUA variability may be suggested. Uric acid emerged as an inflammatory factor that increases oxidative stress [32]. Therefore, varying levels of SUA may be associated with increased oxidative stress, which can contribute to excess CV risk. Another plausible explanation relies on the anti-oxidant role of uric acid. It has been found that uric acid has anti-oxidant effects, suggesting that varying SUA levels might reflect a compensatory mechanism to counter oxidative stress which constitutes a major risk factor for the development of CV disease [16]. It is likely that SUA variability is parallel to variability of cardiovascular risk factors such as hypertension, diabetes mellitus, hyperlipidemia and renal failure [1, 2]. Therefore variation in SUA levels at a relatively young age may reflect development of other CV risk factors, more significantly than persistent hyperuricemia by itself.

The practical implication for the findings in our study is that when assessing SUA levels, consecutive annual measurement should be observed. Patients with varying levels should be followed up more closely for the existence and development of CV risk factors. Our study has several limitations. First, our study was comprised solely of men. Therefore, the conclusion of our study may not apply to women. The findings in our study apply only to a heterogeneous relatively young population. Another limitation is the lack of information regarding drug therapy during follow up which may have affected SUA levels. We were forced to use the SD of SUA-Z score as UA variability measure rather than the SD or coefficient of variance of UA, since we observed a left shift of SUA at 1963 compared to other two observations. We also did not adjust SUA variability to alcohol and diuretic use which can affect SUA levels. However, the rate of alcohol intake and diuretic use were very low. The major strength of our study is the long-term follow up of the cohort. In addition, to the best of our knowledge, this is the first time that clear association between SUA variability and long term CHD and all-cause mortality was assessed. In conclusion, SUA variability may add to the risk assessment of CHD and all-cause mortality.

## Supporting information

S1 File Baseline clinical characteristics, serial and variability of SUA levels and mortality data. (XLS)
